# Longitudinal analysis of influenza vaccination implicates regulation of RIG-I signaling by DNA methylation

**DOI:** 10.1038/s41598-024-51665-9

**Published:** 2024-01-17

**Authors:** Hongxiang Fu, Harry Pickering, Liudmilla Rubbi, Ted M. Ross, Elaine F. Reed, Matteo Pellegrini

**Affiliations:** 1 Department of Molecular Cell and Developmental Biology, University of California Los Angeles, Los Angeles, CA USA; 2 Department of Pathology and Laboratory Medicine, David Geffen School of Medicine, University of California Los Angeles, Los Angeles, CA USA; 3 Department of Infectious Diseases, University of Georgia, Athens, GA USA; 4 Center for Vaccines and Immunology, University of Georgia, Athens, GA USA

**Keywords:** DNA methylation, Vaccines, Biomarkers

## Abstract

Influenza virus infection alters the promoter DNA methylation of key immune response-related genes, including type-1 interferons and proinflammatory cytokines. However, less is known about the effect of the influenza vaccine on the epigenome. We utilized a targeted DNA methylation approach to study the longitudinal effects (day 0 pre-vaccination and day 28 post-vaccination) on influenza vaccination responses in peripheral blood mononuclear cells. We found that baseline, pre-vaccination methylation profiles are associated with pre-existing, protective serological immunity. Additionally, we identified 481 sites that were differentially methylated between baseline and day 28 post-vaccination. These were enriched for genes involved in the regulation of the RIG-I signaling pathway, an important regulator of viral responses. Our results suggest that DNA methylation changes to components of the RIG-I pathway are associated with vaccine effectiveness. Therefore, immunization strategies that target this pathway may improve serological responses to influenza vaccination.

## Introduction

One of the most prevalent pandemic viruses is influenza, which can cause the flu. Influenza occurs worldwide and causes significant morbidity, and its symptoms include fever, cough, sore throat, and nasal discharge. In severe cases, its complications may lead to death^1^. Influenza virus not only affects respiratory organs but also the heart, brain, and muscles^2–4^, and it can be easily spread by respiratory droplets produced from coughing and sneezing^5^. These viruses are also highly mutable. There are two main types of influenza viruses, A and B. The most common influenza A subtypes are H1N1 and H3N2, and the most common influenza B subtypes, known as lineages, are Yamagata and Victoria^6^.

The most effective way to prevent illness and the spread of influenza virus is through annual vaccination. Influenza vaccines induce antibodies against the surface glycoprotein hemagglutinin^7^. The seasonal influenza vaccine can induce HA-specific antibody titers, which can be measured using the hemagglutination inhibition (HAI) assay to quantity functional antibodies that block virus agglutination of turkey erythrocytes.

Pre-vaccination factors, including age, prior infection, vaccination history, immune cell frequencies, and transcriptomic profiles are known to be associated with differential responses to influenza vaccination^8^. However, epigenetic regulation of responsiveness to influenza vaccination is mostly unexplored and may provide insights into mechanisms underpinning the immune response to influenza vaccination.

DNA methylation (DNAm) is one of the most widely studied epigenetic mechanisms. It involves the transfer of a methyl group onto the fifth carbon position of cytosine residues. DNAm is associated with gene regulation and hence many biological processes and diseases^9^. In humans, DNAm appears predominantly in the CpG dinucleotide context. The human genome is not methylated uniformly, and CpG islands are usually hypomethylated^10^. Next-generation sequencing (NGS) methods can be applied to bisulfite-converted DNA to profile DNAm. Whole-genome bisulfite sequencing (WGBS) and targeted bisulfite sequencing (TBS) are two approaches that can be used to measure DNAm levels across CpG sites^11^.

Previous studies have shown that influenza virus infection induces changes in DNAm. One study examined 24 genes known to be involved in the inflammatory response and found that the promoters for 7 genes had significant changes in methylation levels They also observed that strains of influenza viruses with different virulence may affect methylation levels at these promoter regions^12^. Using site-specific mutant knock in mice and region-specific demethylation tools, one study was able to confirm how methylated sites affect interferon I production^13^.

Other studies explored how DNAm affects immunosenescence and how age can affect the response to the influenza vaccine. The comparison of responders and non-responders to the influenza vaccine revealed larger differences in DNAm amongst individuals of older ages, suggesting a larger epigenetic remodeling for older people^14^. Additionally, another study identified methylation sites correlated with the humoral response to influenza vaccination. They demonstrated that methylation sites used for predicting humoral immune responses showed enrichment for binding sites of polycomb-group proteins and the FOXP2 transcription factor^15^.

However, none of the previous studies examined longitudinal changes in DNAm following vaccination. In this study, we first analyzed the association of pre-vaccination methylation levels with seroprotection against multiple influenza strains measured by the hemagglutination inhibition assay. We further performed a longitudinal analysis of DNAm and found significant changes in genes associated with viral response pathways, such as the regulation of RIG-1 signaling. Our approach demonstrates that DNAm profiling provides insights into the serological response to the influenza vaccine.

## Results

### Influenza vaccination cohort demographics and targeted bisulfite sequencing

**Figure 1:**
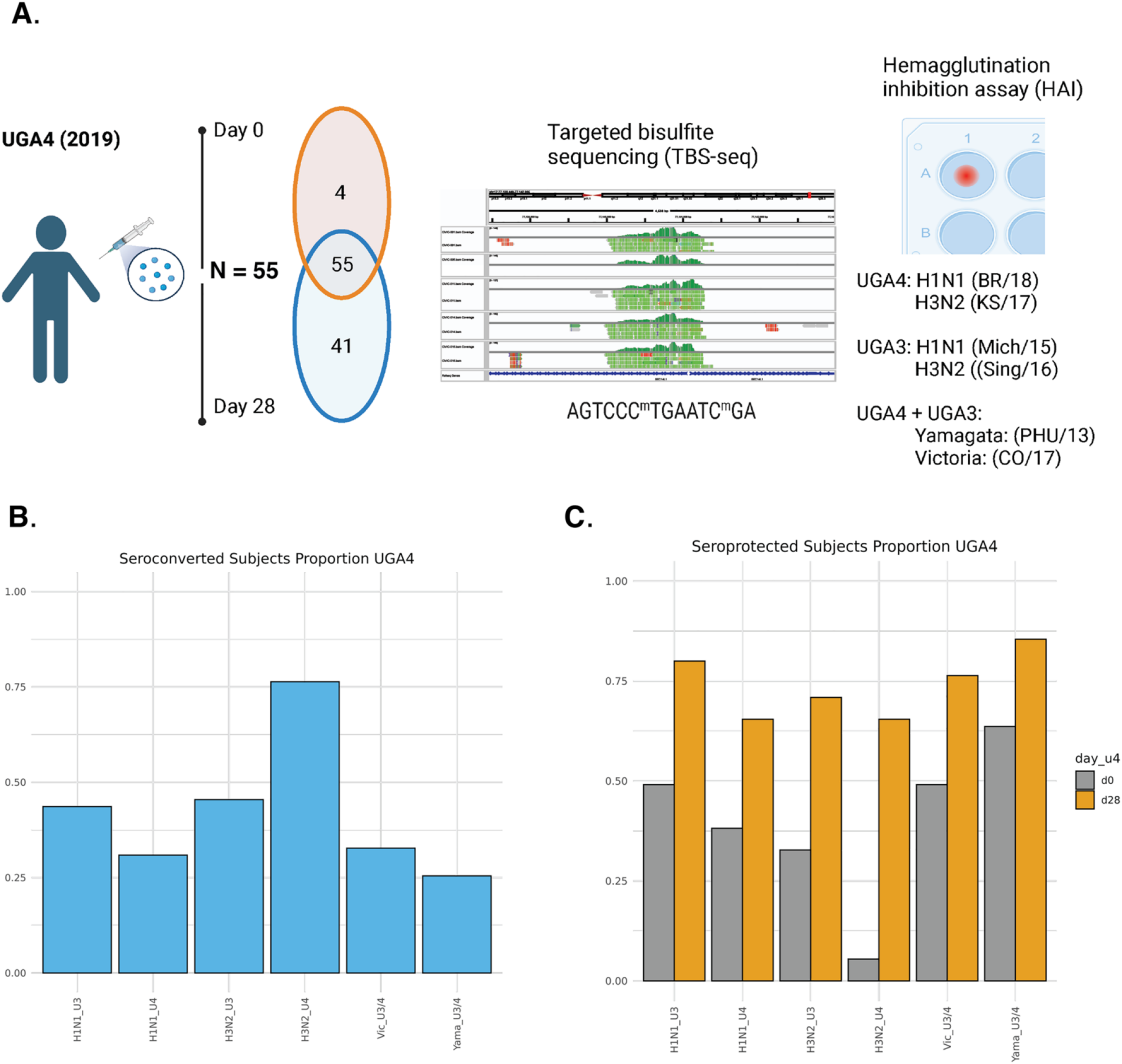
Study design overview. (**A**) Experimental design for this study, 59 subjects at day 0 and 96 subjects at day 28 were vaccinated with licensed Fluzone. Of these participants, 55 were measured on both days. These samples were characterized using targeted bisulfite sequencing (IGV chr17: chr17:77,138,449–77,142,985 SEC14L1) and hemagglutination inhibition assays. (**B**) The proportion of subjects seroconverted 28 days after receiving the vaccination. (**C**) The proportion of subjects seroprotected at day 0 and 28 days after receiving the vaccination**.**

Our study examined cohorts of influenza vaccine recipients enrolled at the University of Georgia from 2016 to 2020^16^, from which we chose in total 55 participants vaccinated in the 2019 cohort (UGA4) who had also previously been vaccinated in the 2018 cohort (UGA3). Participant demographics, as well as pre- and post-vaccination serological immunity to influenza were described in Fig. 1A and Supplementary Table 2 (Additional file 2). The vaccine that was administered was Fluzone, an inactivated, quadrivalent construct consisting of one vaccine substrain from each of the four major subtypes, H1N1, H3N2, Yamagata, and Victoria. For H1N1, the UGA3 vaccine substrain was A/Michigan/45/2015, and UGA4 was A/Brisbane/02/2018. For H3N2, the UGA3 vaccine substrain was A/Singapore/INFIMH-16-0019/2016, and UGA4 was A/Kansas/14/2017. For Yamagata and Victoria, UGA3 and UGA4 included the same vaccine substrains, which were B/Phuket/3073/2013 and B/Maryland/15/2016, respectively. The proportion of the seroprotected and seroconverted samples for each of the UGA3 and UG4 substrains are shown in Fig. 1B and C).

Peripheral blood mononuclear cells were collected at baseline prior to vaccination and 28 days following vaccination. Targeted bisulfite sequencing was performed on 59 individuals for baseline, day 0 (pre-influenza vaccination) and 96 individuals for day 28 (28 days post-influenza vaccination). 55 samples were paired between pre-vaccination and post-vaccination. We sequenced approximately 5000 targeted regions within the genome that were selected based on previous evidence that they were associated with viral infections (Additional file 3: Supplementary Table 3). In total, we quantified levels of DNAm at approximately 70,000 CpG sites. The specific criteria for selecting targeted regions are described in the "Methods" section. BSBolt^17^ was used to align the reads and quantify methylation levels. The final methylation matrix contained 15,631 CpG sites after requiring that each site be covered by at least 20 reads in all the samples as described in detail in the "Methods" section.

### Association of pre-vaccination methylation with seroprotection and seroconversion against influenza vaccine substrains

**Figure 2:**
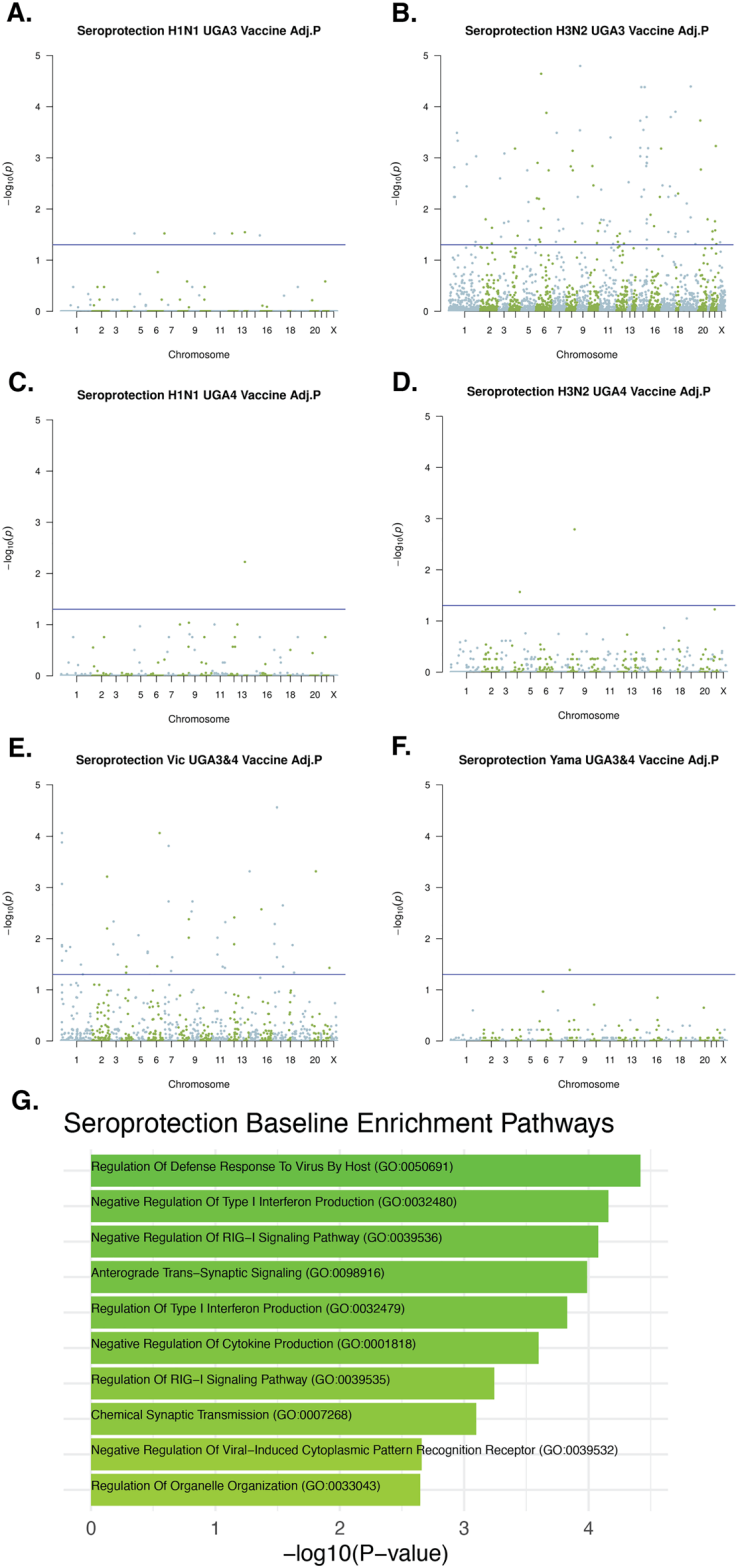
Association of pre-vaccination methylation with day 0 seroprotection of 6 vaccine substrains. Linear regression adjusted by age, sex, and BMI of DNAm with seroprotection of (**A**) H1N1 UGA3 (**B**) H3N2 UGA3 (**C**) H1N1 UGA4 (**D**) H1N1 UGA4 (**E**) Yamagata UGA3&4 (**F**) Victoria UGA3&4 vaccine substrains. The p values were adjusted using the Benjamini–Hochberg procedure. (**G**) Top 10 most significantly enriched pathways that contain genes that are proximal to the union of all significant CpG sites against all targeted background sites.

We first analyzed baseline (day 0) methylation data collected prior to vaccination. Regression analysis adjusted by age, sex, and BMI was performed on each individual CpG site for association with seroprotection at day 0 for the 6 vaccine substrains included in UGA3 and UGA4 (Fig. 2A–F). We found that 121 out of 178 CpG sites that had significant associations with seroprotection (Padj < 0.05) were associated with virus substrains contained only in the Fluzone construct given in UGA3, 54 sites were associated with UGA 3/4 shared strains, and only 3 sites were associated with UGA4 substrains alone.

To investigate potential pathways enriched in CpG sites associated with pre-vaccination seroprotection, we pooled the significant sites for the 6 vaccine substrains and mapped them to proximal genes as described in the "Methods" section to perform a gene set enrichment analysis. The most enriched gene set among the 117 genes we identified was associated with the RIG-I signaling pathway (Fig. 2G).

**Figure 3:**
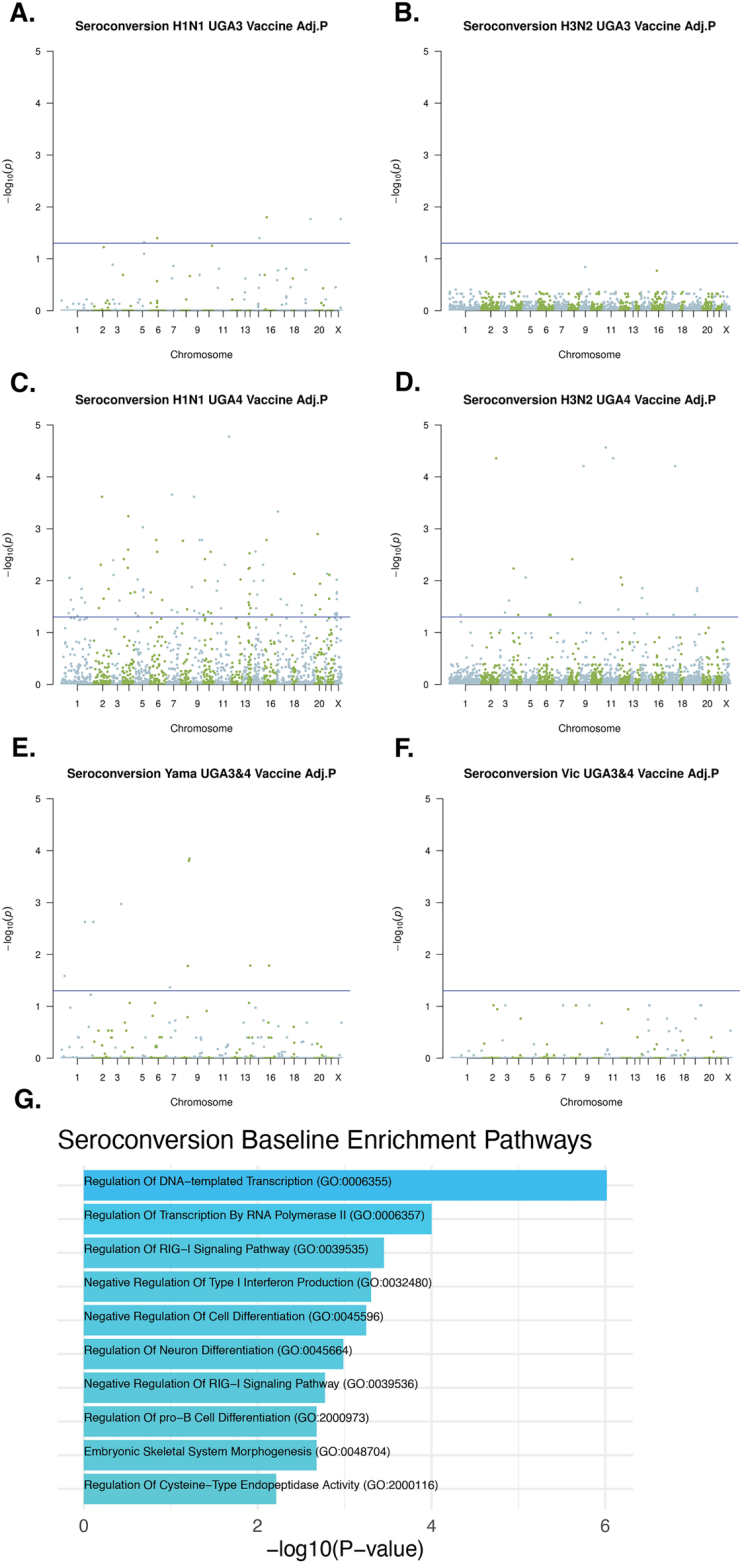
Association of pre-vaccination methylation with seroconversion of 6 vaccine substrains. Linear regression adjusted by age, sex, and BMI of DNAm with seroconversion of (**A**) H1N1 UGA3 (**B**) H3N2 UGA3 (**C**) H1N1 UGA4 (**D**) H1N1 UGA4 (**E**) Yamagata UGA3&4 (**F**) Victoria UGA3&4 vaccine substrains. The p values were adjusted using the Benjamini–Hochberg procedure. (**G**) Top 10 most significantly enriched pathways that contain genes that are proximal to the union of all significant CpG sites against all targeted background sites.

We also carried out a regression analysis to measure the association between baseline DNAm levels and seroconversion at day 28 and investigated the same 6 vaccine substrains (Fig. 3A–F). In contrast to the association with seroprotection, 125 out of 141 significant CpG sites associated with seroconversion were for viruses contained only in the UGA4 vaccine. However, when we carried out an analysis of enriched gene sets associated with genes that were proximal to the significant CpG sites we again found that the most significant enrichment was for the RIG-I signaling pathway (Fig. 3G).

To validate whether DNA methylation in promoter regions have a negative correlation with transcription, we obtained the day 0 pre-vaccination bulk RNA sequencing data for this cohort from a previous study and computed the distribution of the correlation between baseline significant DNA methylation sites and mapped gene expression in the promoter and gene body region^18^. We observed that as expected DNA methylation in the promoter region is negatively associated with gene expression (Additional file 4: Fig. S1A,B). The RIG-I signaling pathway genes GPATCH3, SEC14L1, C1QBP, and NPLOC4 are expressed, and their promoter methylation sites all displayed hypomethylation patterns (Fig. S1C,D).

### Cell type deconvolution at day 0 and day 28 and their correlations with phenotypic traits

**Figure 4:**
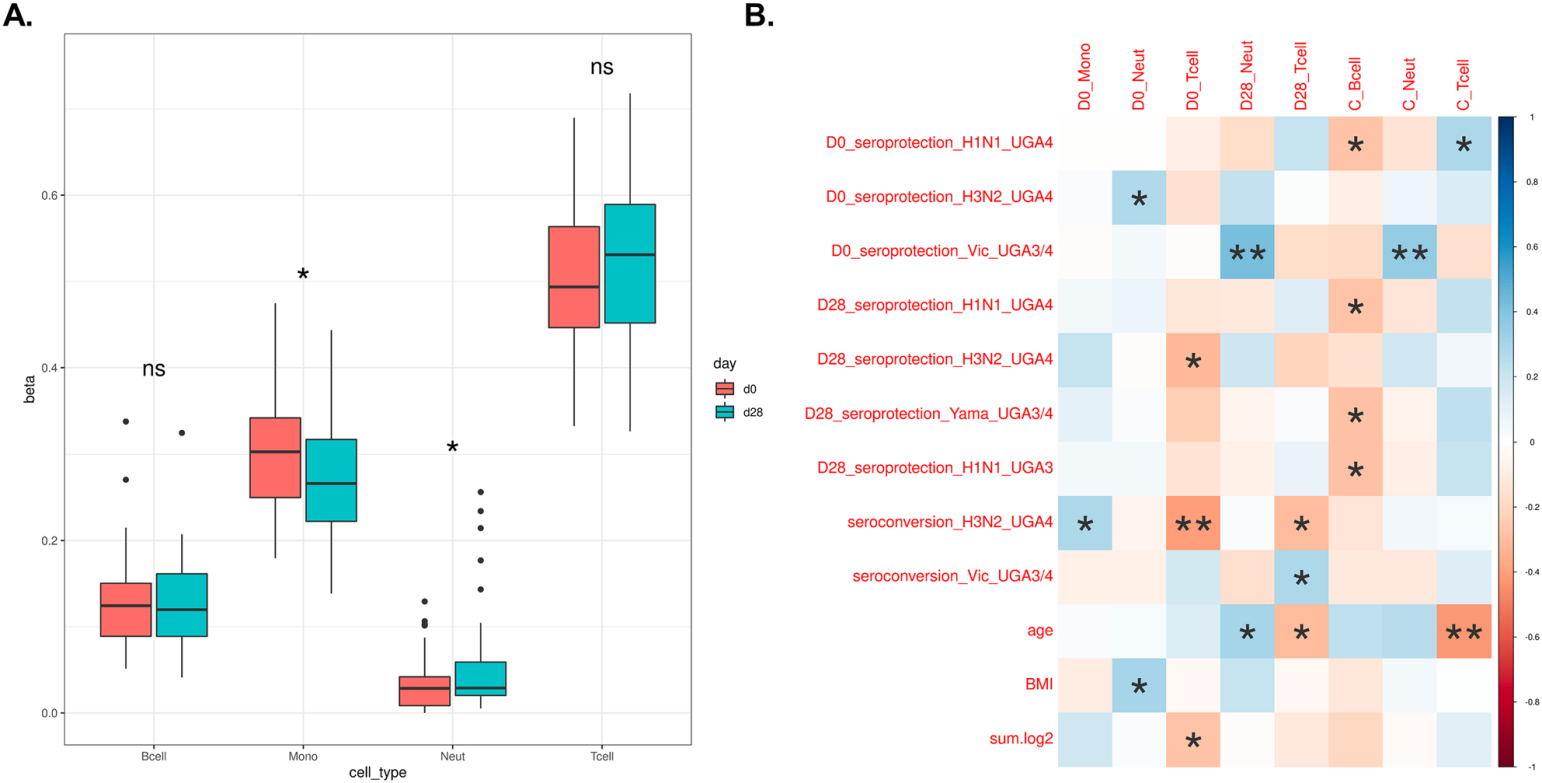
Cell type deconvolution and correlation with phenotypic traits. (**A**) Cell type deconvolution for pre-vaccination and 28 days post-vaccination using methylation profiles. Significance of the changes in abundance was calculated using a two-sided paired t-test. (**B**) The correlation between different cell type proportions and phenotypes. Only significant rows and columns were kept (*Indicates p < 0.05, **Indicates p < 0.01).

To estimate the relative amount of immune cell types contained in each PBMC sample, we carried out cell type deconvolution on both day 0 and day 28 methylation matrices using CellFi^19^. The reference signature matrices contained four main cell types that are found in peripheral blood mononuclear cells (PBMC): B cells, monocytes, neutrophils, and T cells. We found that there was an increase in the proportion of T cells and a decrease in B cells between day 0 and day 28, although these results were not significant. By contrast, we found a significant decrease in monocytes (Fig. 4A). We also calculated the correlation between cell type proportions and seroprotection and seroconversion against the six vaccine substrains (Fig. 4B). The change in cell type proportion was calculated by using the day 28 cell type proportion minus the day 0 cell type proportion. We found that some cell type abundance changes were significantly correlated with some of the variables. For example, the change in T cell abundance was significantly negatively correlated with age. The full correlation heatmap is shown in Fig. S2 (Additional file 4).

### Longitudinal changes in DNA methylation are associated with seroconversion

**Figure 5:**
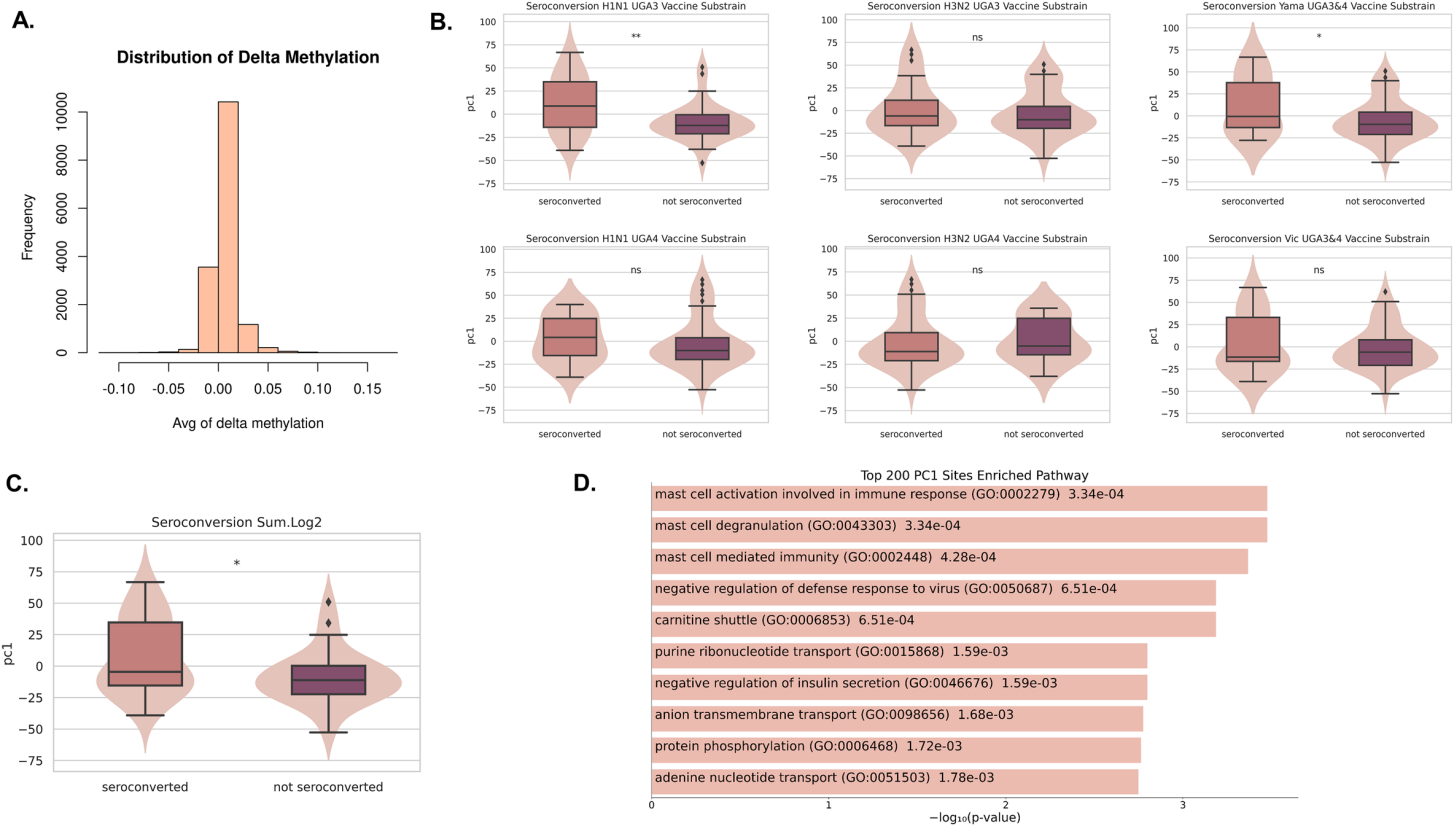
Changes in methylation from day 0 to day 28 associated with seroconversion. (**A**) Distribution of delta methylation from day 0 to day 28. (**B**) Six influenza virus strains show significant differences in the first principal component of the delta methylation matrix between seroconverted and unconverted individuals. (**C**) Summation of Log 2 transformation of the fold change HAI for all six strains show significant differences between seroconverted and unconverted individuals. (**D**) The top ten most significant enriched pathways of the top 200 largest weight sites of the first principal component.

To analyze the change in methylation after vaccination, we subtracted the day 28 methylation values from the day 0 methylation values (the resulting delta methylation distribution is shown in Fig. 5A). Principal component analysis was performed, and we examined whether there were differences in the first principal component (PC1) between the seroconverters and the non-seroconverters for each of the previously selected six vaccine substrains from both UGA3 and UGA4. We found that PC1 values were mostly lower in the non-seroconverted group (Fig. 5B). We computed the sum of the log2 transformation of the fold change HAI for all four UGA4 substrains and split the seroconverters (n = 29) and the non-converters (n = 26) with a cut-off value of 8. We found significant differences in PC1 between the two groups (Fig. 5C). To identify genes that are associated with the changes in PC1 values, we focused on the top 200 CpG sites that had the largest weight of PC1. These sites were then mapped to 70 proximal genes, and then enrichment analysis was performed. The most significantly enriched pathway was associated with mast cell activation (Fig. 5D). One of the genes, PIK3CD, had reduced methylation 28 days post-vaccination, and is believed to affect the maturation of mast cells via the microphthalmia transcription factor^20^.

### Differentially methylation between days 0 and 28 include sites proximal to genes associated with the RIG-I signaling pathway

**Figure 6:**
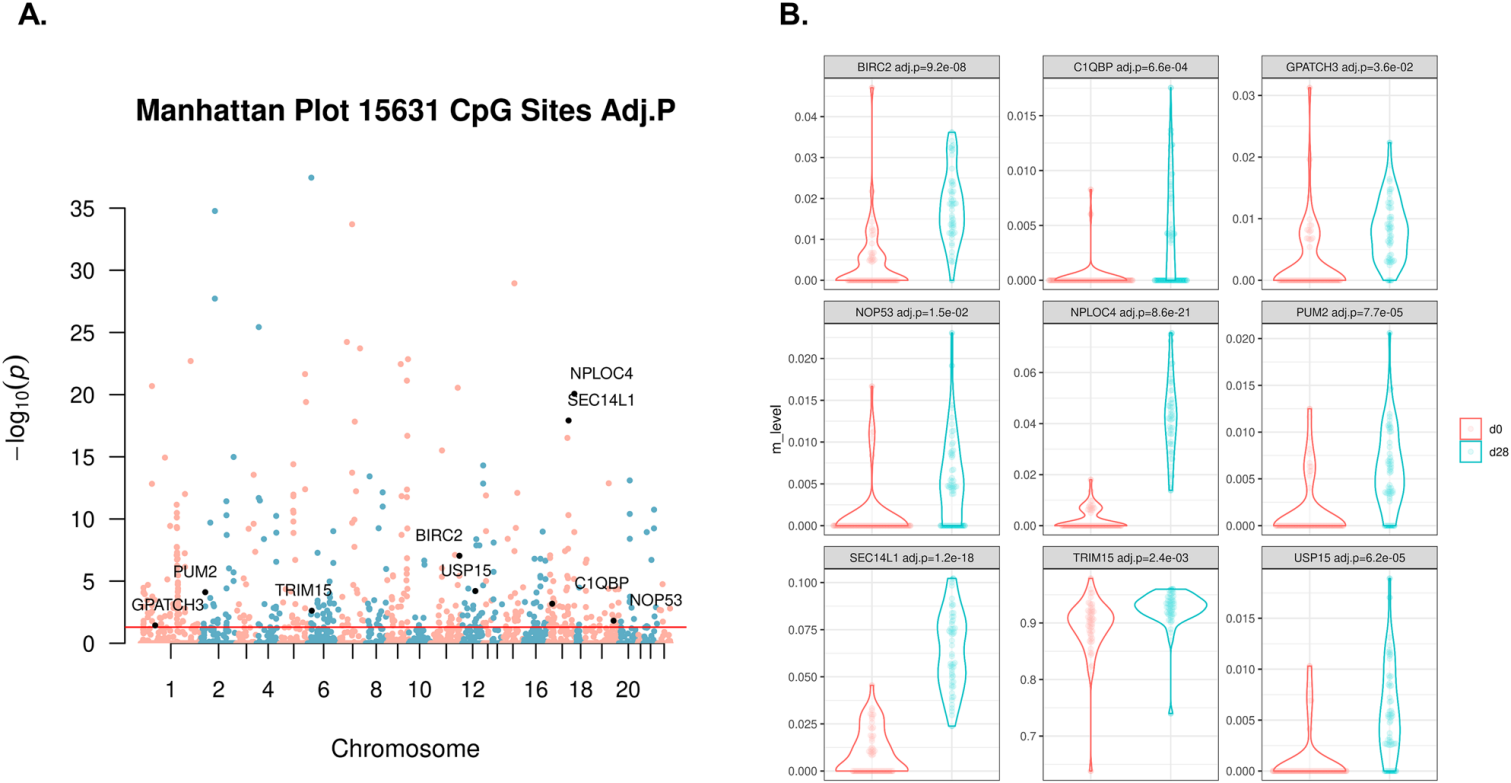
Differential methylation analysis and RIG-I signaling genes. (**A**) Negative log transform of the p-value from the paired t-test, with RIG-I signaling pathway significant genes are highlighted in black. The red line indicates significance based on the Bonferroni correction. (**B**) DNAm level at day 0 and day 28 after vaccination of the 9 significant genes associated with the RIG-I signaling pathway. For each gene, the most significant CpG site was selected when there were multiple sites that map to a gene.

We performed differential methylation analysis on each individual CpG site using a two tailed paired t-test to measure significant changes from day 0 to day 28 (n = 55, and 15,631 CpG sites). There were 481 significantly differentially methylated sites after Bonferroni correction (Fig. 6A). To check if these differentially methylated sites were due to the significant decrease in monocytes, we also conducted a monocyte-specific differential methylation analysis and found no significant sites using CellDMC, which examines the interaction between cell type proportions and the phenotype of interest^21^. We mapped these differentially methylated sites to proximal genes and conducted gene ontology enrichment analysis and reported the top ten most significantly enriched pathways in Table 1. We again found RIG-I signaling to be the most significantly enriched pathway. We show the methylation level changes in the most significant differential methylation site in each of the nine genes that were associated with the RIG-I signaling pathway (Fig. 6B), all of which show an increase in methylation post-vaccination. The second most significant pathway was the regulation of defense response to viruses that includes common influenza virus response genes such as STAT1.

**Table 1:** Top ten enriched pathways from differential methylation analysis.

Pathway	Overlap	Genes	P.value	Adjusted P value
Regulation of RIG-I signaling pathway (GO:0,039,535)	9/19	USP15; NPLOC4; SEC14L1; C1QBP; TRIM15; GPATCH3; NOP53; BIRC2; PUM2	2.994E − 14	4.584E − 11
Regulation of defense response to virus by host (GO:0,050,691)	9/36	RNF216; DTX3L; SIN3A; STAT1; TNFAIP3; EIF2AK4; PARP9; TARBP2; PQBP1	2.672E − 11	2.045E − 08
Regulation of defense response to virus (GO:0,050,688)	7/16	ILRUN; RNF216; ELMOD2; RNF26; C1QBP; PCBP2; TNFAIP3	4.912E − 11	2.506E − 08
Negative regulation of type I interferon production (GO:0,032,480)	9/43	ILRUN; PYCARD; NLRX1; NPLOC4; RNF216; PCBP2; TNFAIP3; SIRPA; GPATCH3	1.515E − 10	5.801E − 08
Regulation of type I interferon production (GO:0,032,479)	10/89	ILRUN; NLRX1; NPLOC4; RNF216; RNF26; PCBP2; TNFAIP3; TRIM15; GPATCH3; PQBP1	8.300E − 09	2.541E − 06
Negative regulation of viral-induced cytoplasmic pattern recognition receptor signaling pathway (GO:0,039,532)	5/10	NPLOC4; SEC14L1; TKFC; C1QBP; GPATCH3	1.434E − 08	3.659E − 06
Negative regulation of defense response to virus (GO:0,050,687)	5/11	ILRUN; C1QBP; RNF26; PCBP2; TARBP2	2.610E − 08	5.708E − 06
Negative regulation of cytokine production (GO:0,001,818)	12/182	PYCARD; ILRUN; NLRX1; NPLOC4; RNF216; C1QBP; PCBP2; SIRPA; TNFAIP3; GPATCH3; ZFPM1; JAK3	1.113E − 07	2.131E − 05
Positive regulation of defense response to virus by host (GO:0,002,230)	6/28	DTX3L; SIN3A; STAT1; EIF2AK4; PARP9; PQBP1	1.667E − 07	2.837E − 05
Negative regulation of RIG-I signaling pathway (GO:0,039,536)	4/7	NPLOC4; SEC14L1; C1QBP; GPATCH3	2.272E − 07	3.479E − 05

Significant methylation sites were mapped to proximal genes either in the gene body or promoter regions. Then significant genes were selected, and gene ontology enrichment analysis were conducted.

### Overlap of genes associated with seroprotection and seroconversion

**Figure 7:**
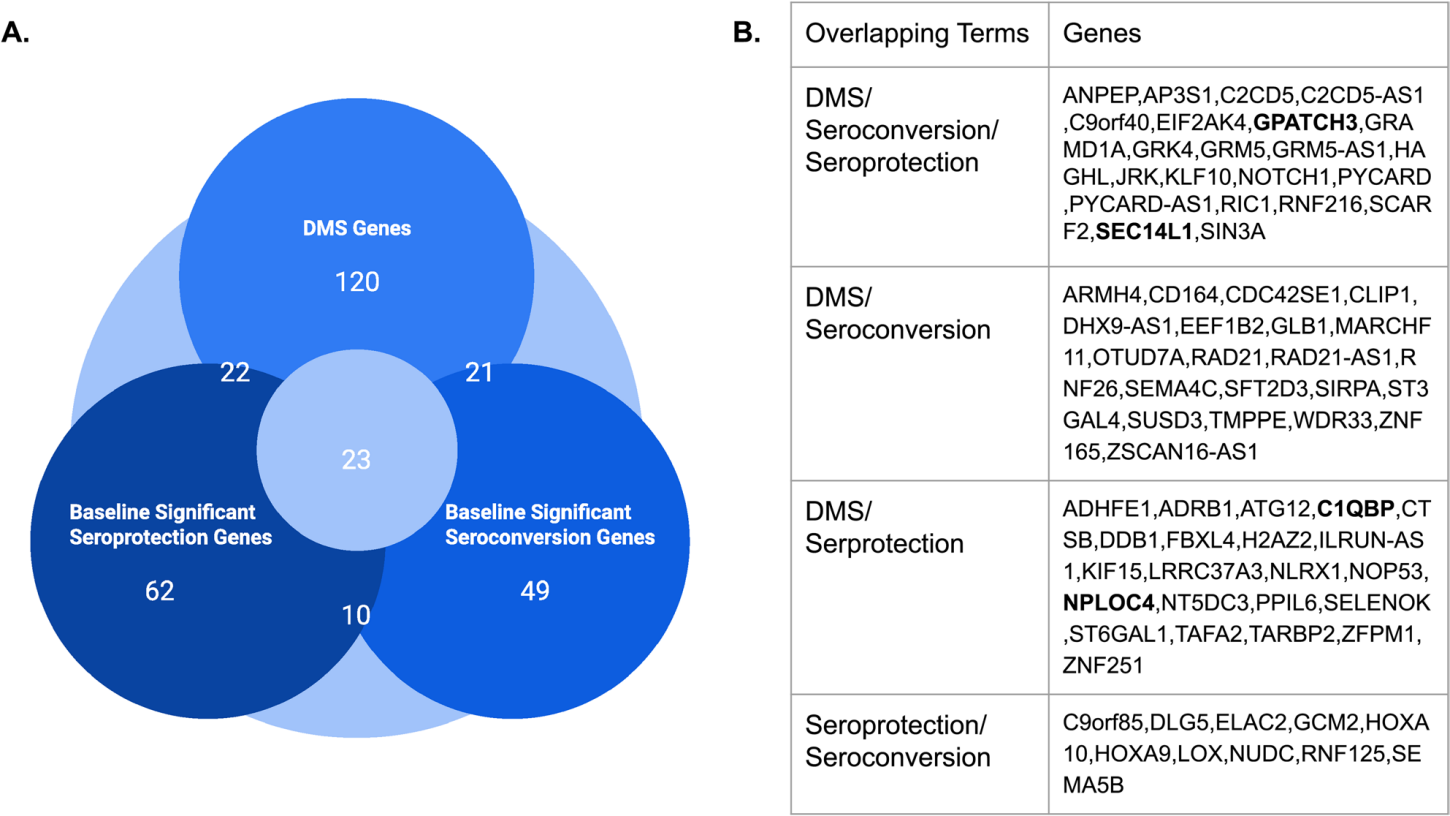
Joint analysis of DMS and baseline inspection. (**A**) Venn diagram of significant DMS genes, baseline seroprotection genes and baseline seroconversion genes. (**B**) A table summarizing all the overlapping genes between each of the three categories.

We combined genes whose methylation was associated with baseline seroprotection, baseline seroconversion, and differentially methylated sites (DMS) between day 0 and 28. We compared the genes identified from each analysis to explore the overlap between these genes and pathways (Fig. 7). Twenty-three genes appeared in all three analyses, and two of them, SEC14L1 and GPATCH3, are involved in negative regulation of RIG-I signaling. Among the overlapping genes between the list of differentially methylated genes and day 0 seroprotection genes, C1QBP and NPLOC4 were also associated with negative regulation of RIG-I signaling. Many other overlapping genes, such as RNF125, have known roles in antiviral immune responses, including the production of type-1 interferons.

## Discussion

In this study, we explored the effect of epigenetic regulation on serological responsiveness to influenza vaccination. Study subjects received the quadrivalent Fluzone vaccine one year prior to sampling for this work (UGA3) and in the study year (UGA4) received an updated construct of Fluzone incorporating the same Yamagata and Victoria Strains, but different H1N1 and H3N2 strains. Pre-vaccination DNAm profiles were associated most strongly with seroprotection against strains only included in Fluzone from UGA3 from the previous year, whereas changes in DNAm post-vaccination were associated with seroconversion against all four strains included in the most recent Fluzone from UGA4. All analyses highlighted epigenetic regulation of RIG-1 signaling, and downstream type-1 interferon responses, as key components of serological response to influenza vaccination.

We first examined pre-vaccination methylation as it provides information about the starting immune state of the individuals. We investigated the association of baseline seroprotection of six vaccine substrains found in UGA3 and UGA4 vaccines with baseline methylation. The majority of significant CpG sites were associated with substrains contained in the UGA3 vaccine which was administered the previous year. The H3N2 and Victoria strains from the UGA3 cohort had more associations with the H1N1 and Yamagata strains. A potential explanation is that the H3N2 and Victoria strains were newly introduced in the UGA3 vaccine, whereas the H1N1 and Yamagata strains were the same as those used in the previous 2017 quadrivalent influenza vaccine. This suggests that there is epigenetic memory associated with the influenza strains from prior vaccinations. This may help explain why vaccination history and pre-existing immunity have consistently been identified as predictive of response to vaccination^16,22,23^, as epigenetic changes from these prior exposures likely persist even if antibody titers wane.

We next examined the association of seroconversion for the same vaccine substrains and their association with baseline methylation. We found that baseline methylation is associated with seroconversion (a fold change in HAI from day 28 to day 0) for the UGA4 substrains but not for UGA3. This suggests that the epigenome at baseline is primed to respond to new viral substrains, and that to some degree the epigenetic profile of an individual can forecast whether that individual will seroconvert against the strains found in the vaccine. We also mapped the significant CpG sites for both seroprotection and seroconversion to proximal genes and conducted gene ontology enrichment analysis. Interestingly, both results identified the regulation of the RIG-I signaling pathway as most significant, which indicates that this pathway is associated with both baseline protective immunity and immune conversion.

We then analyzed the cell type changes after vaccination. We found that there was a slight increase in the proportion of T cells and a slight decrease in B cells, and significant changes in monocytes. Neutrophil levels were low as we expected from PBMC samples, which typically exclude the neutrophil fraction. We computed the correlation between the change in cell types and different variables. Our findings that the change in T cells was negatively correlated with age were consistent with previous studies, which suggested that T cell activation becomes weaker with age^24^. This result is consistent with the observation that response to vaccination wanes with age^25^. The observed significant alterations in the relative proportion of monocytes are suggestive of a transition from the innate to the adaptive immune response, which is essential for the induction of protective immunity memory following vaccination. This induction process is mediated by the production of interferons^26^.

Finally, we investigated the change in methylation between baseline and day 28 in relation to protective immunity and immune conversion. We showed that when analyzing changes in DNAm by principal component analysis, the first principal component (PC1) value tended to be lower in the seroconverted group, suggesting there is a global association between longitudinal changes in DNAm and seroconversion. We next sought to identify the sites that contributed most strongly to PC1 and found that the genes mapped to the top 200 sites were enriched in mast cell activation involved in immune responses. Our finding supports previous work that demonstrated that mast cells influence the response to influenza virus infection in mice^27^. We also mapped significant differentially methylated CpG sites post-vaccination to proximal genes and performed gene ontology enrichment analysis. Again, we find that the RIG-I signaling pathway is associated with longitudinal changes in methylation. Four genes coinciding with baseline seroprotection and seroconversion following vaccination were identified as negative regulators of the RIG-I signaling pathway. An increase in methylation within their promoter regions suggests a downregulation of gene expression, thereby potentially enhancing the activation of the RIG-I signaling pathway. This observation is supported by previous studies that have documented the role of RIG-I pathway activation in enhancing immune responses post-influenza vaccination^28^.

**Figure 8:**
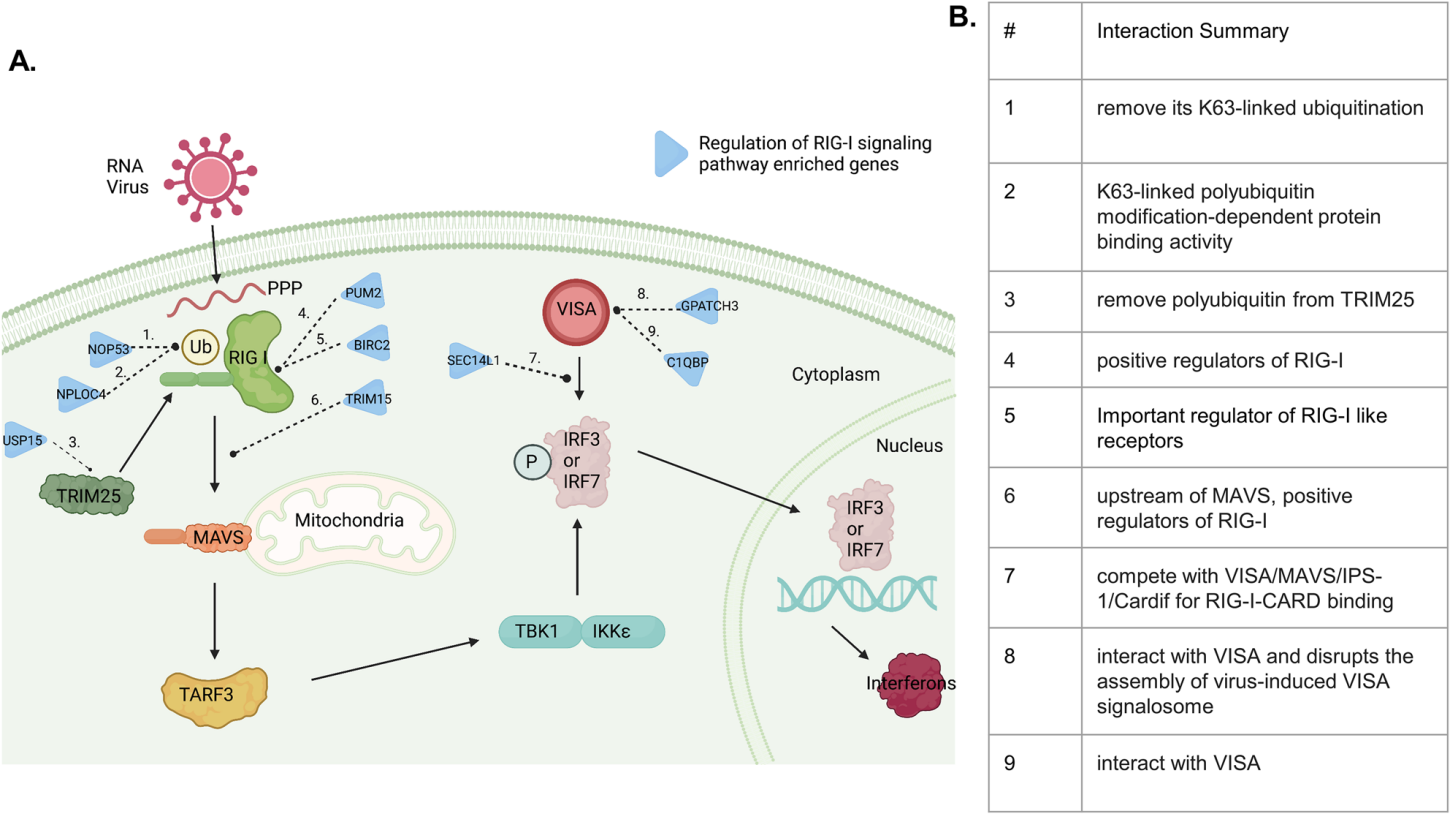
RIG-I pathway summarization with significant differential methylated genes. (**A**) Generated using BioRender, the full pathway of RIG-I signaling starting from virus detection to interferon production. (**B**) A summary table of each of the genes’ functions.

The RIG-I signaling pathway was previously shown to be involved in antiviral responses^29,30^. Following viral infection, the RIG-I signaling pathway drives the transcription of type-1 interferons, which normally are expressed at low and undetectable levels. Type-1 interferons are a key component of innate immune defense against viral infection, while also promoting initial adaptive immune responses. However, they can also cause immunopathology in acute viral infections, and can lead to immunosuppression and loss of virus control during chronic viral infections^31^. The genes and DNA methylation CpG sites within the RIG-I signaling pathway and how they contributed to immune response remains to be fully characterized. We identified nine RIG-I signaling genes proximal to differentially methylated sites and investigated their functions based on previous studies and their interactions are depicted in Fig. 8.

NOP53 is a tumor suppressor protein that translocates to the cytoplasm during viral infection. It was previously found to remove K63-linked ubiquitination, which leads to the decreased transcription of type 1 interferons^32^. NPLO4 is involved in K48-linked polyubiquitin modification-dependent protein binding activity^33^, which also negatively regulates the production of type 1 interferons. USP15 is a positive regulator of the RIG-I-mediated antiviral immune response and removes polyubiquitin from TRIM25 which promotes its stabilization and enhancement of RIG-I viral responses^34^. PUM2 is a translational repressor involved in cytosolic viral sensing through LGP2, which itself stimulates type-1 interferon production while regulating RIG-I signaling^35,36^. BIRC2 is an E3 ubiquitin-protein ligase; it functions as a repressor of the NF-kB pathway^37^, which itself is positively regulated by RIG-1 signaling^38^. TRIM15 is a RIG-I-like receptor protein regulator required for RIG-I signaling and downstream type-1 interferon production^32,39^. SEC14L, GPATCH3 and C1QBP compete or interact with virus-induced signaling adaptor molecules (VISA), and can disrupt the assembly of virus-induced VISA signalosomes which negatively regulate the RIG-I signaling pathway^40–43^.

Taken together, these functions suggest epigenetic regulation of RIG-I signaling, and related pathways that control type-1 interferon production, is an important component of influenza vaccine responses. In support of this, adjuvants that activate RIG-I signaling can enhance both serological and cellular response to influenza vaccination^44–46^. While the exact mechanisms behind this effect are incompletely understood, induction of type-1 interferons is consistently identified as a key factor. Future research may be directed toward elucidating the mechanisms by which DNA methylation modifications enhance the specificity of immune responses to distinct viral antigens. Furthermore, investigations could explore the dynamic relationship between the innate and adaptive branches of the immune system and how these are moderated by DNA methylation patterns. Insights from such studies have the potential to contribute to the development of more potent and durable immune responses.

## Conclusions

In conclusion, we identified differential methylated sites post-vaccination and baseline methylation profiles associated with protective immunity prior to and following influenza vaccination. Consistent patterns of DNAm were detected near genes involved in the regulation of the RIG-I signaling pathway, suggesting that vaccination constructs and/or strategies that enhance activation of this pathway may be beneficial. Our finding also suggests that mast cell activation involved in immune responses could play a role in affecting seroconversion. Finally, certain immune cell type proportions and cell type changes were correlated with the immune response to the influenza vaccine. We find that the epigenome allows us to identify pathways involved in the regulation of responsiveness to influenza vaccination.

## Material and methods

### Cohorts of seasonal influenza vaccination

The cohorts we examined have been described in detail previously^16^. Briefly, as part of an ongoing study by the University of Georgia, Athens (UGA), a total of 690 participants were recruited during five seasons between 2016 and 2020 (UGA1-5). The study procedures, informed consent, and data collection documents were reviewed and approved by the Institutional Review Board of the University of Georgia (IRB #3773). All methods were performed in accordance with the relevant guidelines and regulations. Participants received the split-inactivated influenza vaccine Fluzone by Sanofi Pasteur. For the 2018–2019 season (UGA3), the strains were A/Michigan/15 (H1N1), A/Singapore/16 (H3N2), B/Phuket/2013 (Yamagata lineage), and B/Colorado/2017 (Victoria lineage). For the 2019–2020 season (UGA4), the strains were A/Brisbane/2018 (H1N1), A/Kansas/2017 (H3N2), B/Phuket/2013 (Yamagata lineage), and B/Colorado/2017 (Victoria lineage). Participants provided blood samples on the day of vaccination (Day-0) prior to vaccine administration, and 7/28 days post-vaccination (Day-7 and Day-28). Hemagglutination inhibition (HAI) assays were performed on the Day-0 and Day-28 blood samples against each of the vaccine strains as well as other strains as described in detail in the previous study^16^. For this study, we selected and analyzed a subset of 55 participants who were enrolled and vaccinated as part of UGA3 and remained enrolled at both time points of UGA4. In this paper, we define seroprotection as the HAI titer and seroconversion as the HAI titer fold change compared to the baseline. A subject is seroprotected if the HAI titer ≥ 1:40, and a subject is seroconverted if the HAI titer fold change ≥ 4.

### Targeted bisulfite sequencing (TBS-seq)

#### Probe selection

The selection of targeted bisulfite sequencing (TBS-seq) CpG probes is based on the following criteria. First, they have been selected to be used in DNA methylation-based aging clocks^47^. Second, they are located in the promoters (− 1000 bp/ + 250 bp from tss) of viral-response genes associated with SARS and influenza infections^48–51^. Third, they have blood and immune cell-type-specific DNAm profiles built using CellFi reference from the Blueprint Epigenome Project^19,52^. Fourth, they have associations with body metabolism phenotypes such as BMI^53,54^. The probes that cover the selected CpG sites have been synthesized by IDT, and the coordinates (GRCH38) of the criteria are listed separately in Supplementary Table 3 (Additional file 3).

#### Library preparation

Using the standard phenol–chloroform extraction method for blood samples as described in this paper, DNA was isolated from each individual^55^. For the TBS-seq library preparation, 500 ng of extracted DNA were used^56^. Adapter ligation and dA-tailing were performed using the NEBNext Ultra II Library prep kit and custom pre-methylated adapters (IDT)^57^. Purified libraries were then hybridized to the biotinylated probes. Under the following conditions: 2 min at 98 °C; 14 cycles of (98 °C for 20 s; 60 °C for 30 s; 72 °C for 30 s); 72 °C for 5 min; hold at 4 °C. PCR amplification was performed after bisulfite treatment on captured DNA using KAPA HiFi Uracil +. Library quality control was conducted on the high-sensitivity D1000 assay on a 2200 Agilent TapeStation. Two NovaSeq6000 (Sp lane) were used to sequence the libraries as 150 paired-end bases.

### Data processing and methylation calling

Adapter sequences were removed using cutadapt (v2.10) for the demultiplexed FastQ files^58^. Then, BSBolt (v1.3.0) was used to align the reads to the reference GRCh38 genome^17^. Samtools was used to remove PCR duplicates using the markdup function^59^. Lastly, methylation calling was performed using the BSBolt CallMethylation function. Methylation values were calculated by taking the sum of methylated cytosine and unmethylated cytosine, and then dividing by the coverage. For the final step of generating the methylation matrix, all the CpG sites need to be covered by at least 20 reads in all the samples using the AggregateMatrix function in BSBolt. This work used computational and storage services associated with the Hoffman2 Shared Cluster provided by UCLA Institute for Digital Research and Education’s Research Technology Group.

### Statistical testing

#### OLS regression analysis

Ordinary least square (OLS) regression analysis was performed on the day 0 methylation matrix for both cohort 3 and cohort 4 vaccine substrains. And both seroprotection (HAI score at day 0) and seroconversion (HAI score fold change from day 28 to day 0) were regressed against individual CpG sites. Benjamini–Hochberg FDR method was used to adjust for multiple testing problems. And the final p values for testing if the coefficients are zero or not was log transformed for data visualization.

#### Delta methylation and principal component analysis

Delta methylation matrix was obtained from the change of methylation matrix from day 28 to day 0. The values of delta methylation were standardized before performing the principal component analysis (PCA). PCA was then conducted for the standardized delta methylation matrix using the scikit-learn package^60^. The first principal component explains the most variance, and the 200 top sites of the first principal component were selected by looking at their weights to the first pc which can be both positive and negative values.

#### Paired t-test and differentially methylated sites (DMS)

Two sided paired t tests were performed for each individual CpG site at day 28 and day 0. The differentially methylated sites were selected based on p-value from the t test result. Those sites (481 sites) that passed the significance threshold 0.05 after Bonferroni correction were considered significant.

### Cell type deconvolution for methylation matrix

Four main blood cell types were used as references when performing cell composition estimation using CellFi which are neutrophils, T cells, B cells and monocytes. The reference datasets were obtained from the Blueprint Epigenome Project^52^. A non-negative least squares regression was performed on the methylation level of cell type specific regions of the samples and references to estimate the cell type proportion for each sample.

### Proximal genes and gene set enrichment analysis

Each CpG site was mapped with the nearest gene features using the Biomart package (v2.28.0) in R (4.1.0)^61^. Proximal genes were defined by the criteria that the site was either within the gene body or in the promoter region that is in the range of -1000 to + 250 base pairs away from the transcription start site. The dataset homo sapiens genes (GRCh37.p13) was used to implement the CpG to gene annotation. Then, the gene set enrichment analysis was performed using Enrichr against all targeted background sites and verified using GREAT (Additional file 4: Fig. S3)^62,63^. The gene ontology biological process was used as the gene set, and a hypergeometric test was carried out to conduct the gene set enrichment analysis.

### Ethics approval and consent to participate and publish

This study has been approved by the University of Georgia Institutional Review Board. Written consent forms for participation were obtained from all participants, their parents, or their legal guardians. Written consent forms that approve publication were obtained from all participants, their parents, or their legal guardians.

### Supplementary Information

Supplementary Table 1.

Supplementary Table 2.

Supplementary Table 3.

Supplementary Figures.

## Data Availability

All the raw sequence FastQ data, processed methylation CGmap data, and metadata are uploaded to Gene Expression Omnibus (GEO) and are accessible through GEO series accession number GSE215157.

## Abbreviations

DNAm: DNA methylation
NGS: Next-generation sequencing
WGBS: Whole-genome bisulfite sequencing
TBS: Targeted bisulfite sequencing
HAI: Hemagglutination inhibition assay
UGA: University of Georgia cohorts
PMBC: Peripheral blood mononuclear cells
DMS: Differential methylated sites
PC: Principal component
GEO: Gene expression omnibus
