# Supplementary material for: Longitudinal analysis of influenza vaccination implicates regulation of RIG-I signaling by DNA methylation
Source: Sci Rep. 2024 Jan 17;14:1455. doi: 10.1038/s41598-024-51665-9 (PMC10791625; doi:10.1038/s41598-024-51665-9)
Supplement: Supplementary file 4 — Supplementary Figures. [file 41598_2024_51665_MOESM4_ESM.pdf]

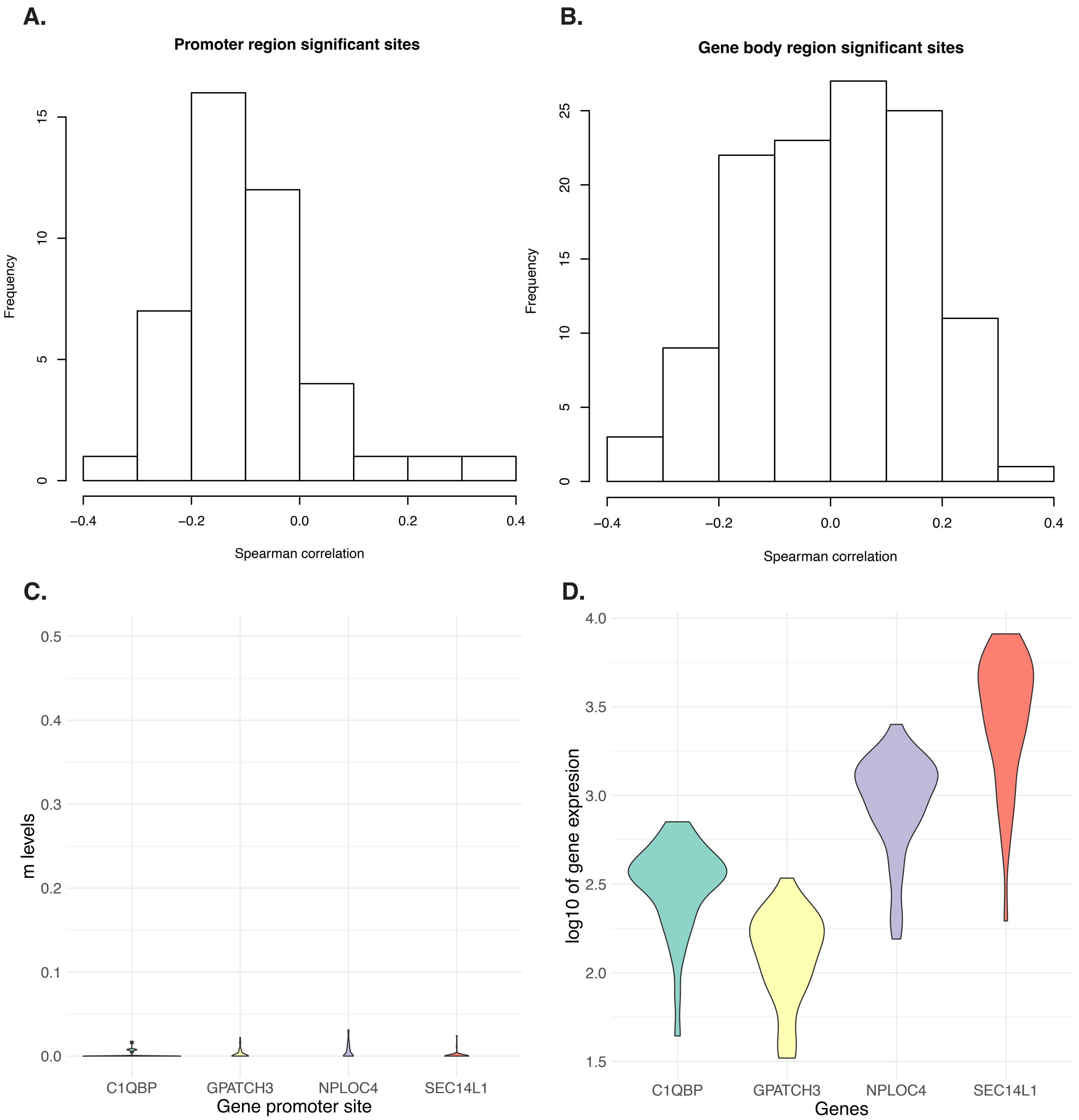

Figure S1. Integration of day 0 gene expression with day 0 methylation. (A) Spearman correlation of baseline significant methylation sites with mapped genes in the promoter region. (B) Spearman correlation of baseline significant methylation sites with mapped genes in the gene body region. (C) DNA methylation level at day 0 of the promoter region for the four negatively-regulated RIG-I genes. (D). Log10 gene expression level at day 0 of the four negatively-regulated RIG-I genes.

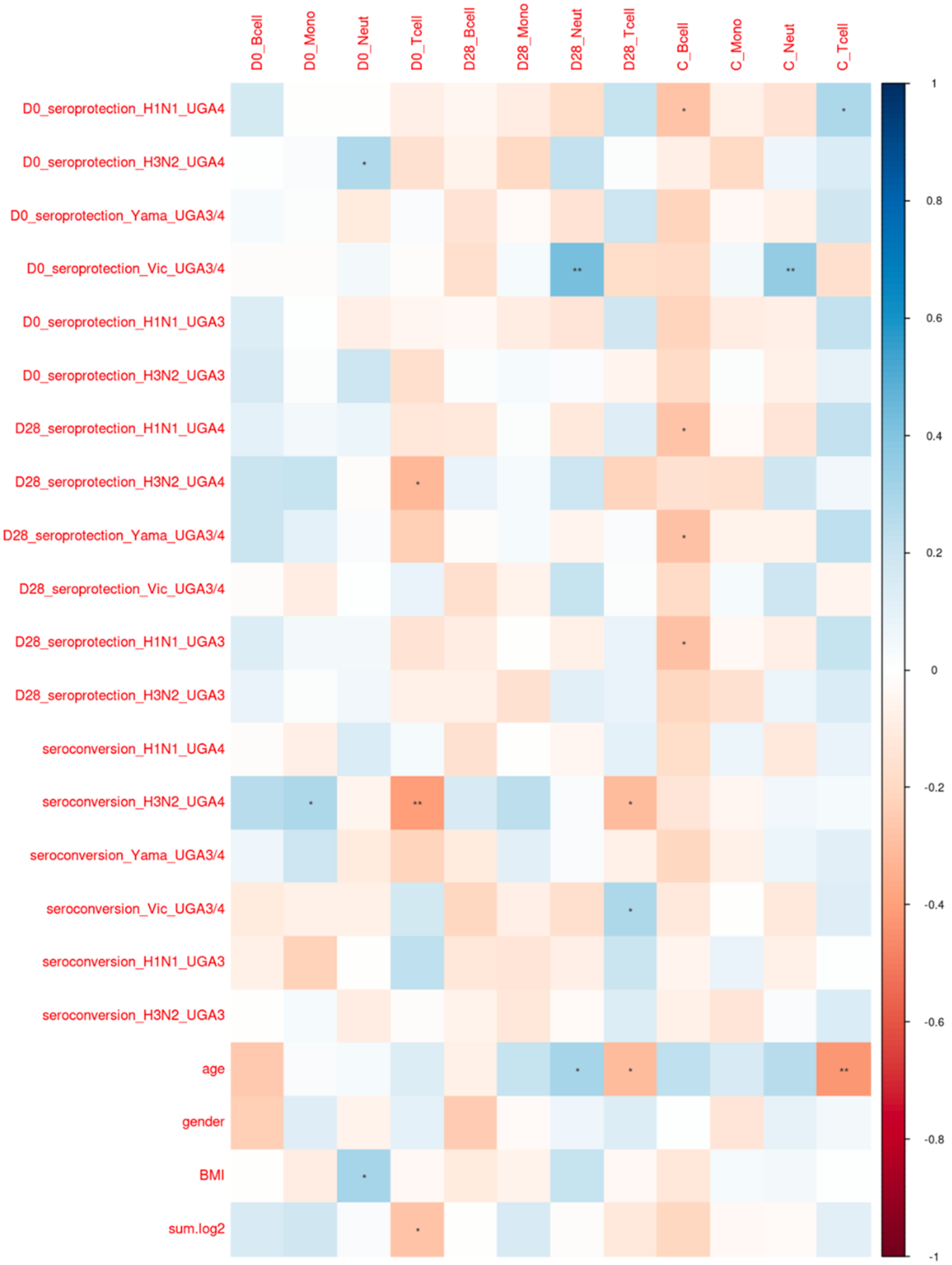

Fiure S2. Full cell type correlation with phenotypic traits. The full correlation heatmap between different cell type proportions and phenotypes.

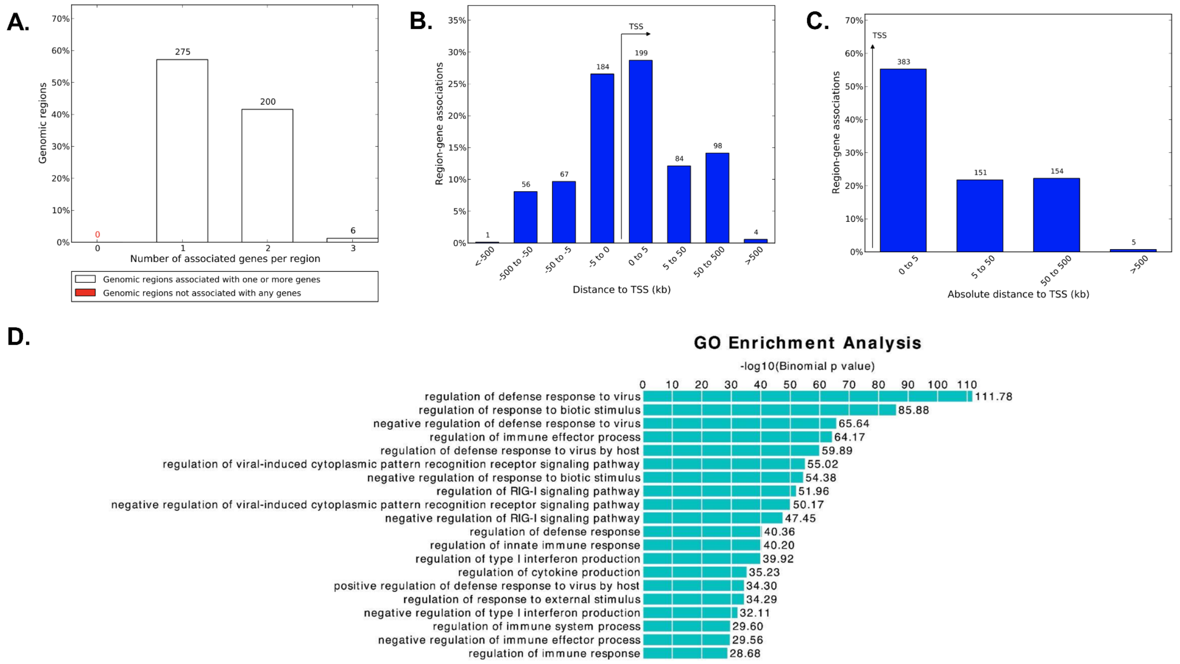

Figure S3. Top twenty enriched pathways from differential methylation analysis. Significant methylation sites were mapped to proximal genes and gene ontology enrichment analysis were verified using GREAT.
